# Expression systems for bovine rhodopsin: a review of the progress made in the Khorana laboratory

**DOI:** 10.1007/s12551-022-01037-2

**Published:** 2023-01-06

**Authors:** Philip J. Reeves

**Affiliations:** School of Life Sciences, University of Essex, Wivenhoe Park, Colchester, CO4 3SQ Essex UK

**Keywords:** G protein-coupled receptors, Membrane proteins, Glycosylation, Tetracycline-inducible, Sodium butyrate

## Abstract

Here I will review the development of gene expression systems for production of bovine rhodopsin in the Khorana laboratory with particular focus on stable mammalian cell lines made using human embryonic kidney cells (HEK293S). The synthesis of a gene encoding bovine rhodopsin was completed in 1986. This gene was expertly designed with the built-in capacity for DNA duplex cassette replacement mutagenesis which made site-directed mutagenesis relatively straightforward. Intense effort was expended over several years in order to identify a gene expression system capable of producing rhodopsin in milligram amounts as required for biophysical studies. Mammalian expression systems, both transient and stable, were found to be the most favourable based on several criteria including receptor expression levels, correct folding and post translational processing, and capacity for purification of fully functional receptor. Transient expression using COS-1 cells was preferred for routine small-scale production of rhodopsin mutants, while HEK293S stable cell lines were used when milligram amounts of rhodopsin mutants were needed; for example, when conducting NMR studies.

## Early challenges in gene expression in the Khorana laboratory

Challenges associated with gene expression were often encountered during Gobind Khorana’s research career. The Khorana group was responsible for the construction of the first fully synthetic genes, one of which was a naturally occurring *Escherichia coli* suppressor tRNA for tyrosine. Synthesis of this gene was completed in the mid-1970s. However, an understanding of the factors controlling expression (promoter and termination sequences) of genes was not fully understood at that time. The uncertainty surrounding this potential problem did not deter Khorana from initiating this project. As predicted by Khorana, such information would in due course become available, thus allowing expression of this gene (Caruthers 2012). A similar challenge facing Khorana was encountered when his research effort was re-focussed to bacteriorhodopsin (bR). Strand synthesis site-directed mutagenesis was problematic at this time when applied to the bR gene (bop) (Karnik et al. 1987) so a fully synthetic bop gene containing regularly spaced unique restriction enzymes was constructed (Nassal et al. 1987). This design enabled site-directed mutagenesis by synthetic duplex DNA cassette replacement. A further complication was that expression systems for the natural host of the bop gene (*Halobacterium halobium*) were unavailable at that time. For this reason, the synthetic bop gene was cloned into a vector for recombinant expression in *E. coli* (Nassal et al. 1987). However, bR thus produced was misfolded. The problem of re-folding denatured bR was solved (Huang et al. 1981), which enabled the biochemical and biophysical examination of site directed bR mutants. The ability to fold denatured bR present in *E. coli* inclusion bodies to a native form was a remarkable feat indeed. Furthermore, this progress additionally provided new ways to examine fundamental aspects of protein folding such as kinetics of folding and ligand binding. Gobind’s unique knowledge in this discipline allowed us to overcome a related problem we encountered during our effort to stabilise mammalian rod opsin apoprotein for use in studies to probe the binding of its ligand 11-*cis* retinal (Reeves et al. 1999).

Many milligrams of misfolded bR could now be obtained, especially after scale-up of production using growth in a bacterial fermenter (Stern and Khorana 1989). Eventually, transgene technology was developed for expression of bR mutants using the natural host (*Halobacterium halobium*) which simplified their large-scale expression (Krebs et al. 1991). The capability for making milligram amounts of bR mutants needed for biophysical analysis proved pivotal for understanding the molecular mechanism of light-mediated proton pumping in bR (Khorana 1993).

## Expression of bovine rhodopsin in the Khorana laboratory—an overview

In the mid-1980s Khorana also switched attention to the study of mammalian rhodopsin (Rho). Many of the tools developed previously in the laboratory were deployed including the design and construction of a synthetic gene encoding Rho containing regularly spaced restriction enzyme recognition sites to facilitate cassette replacement mutagenesis (Ferretti et al. 1986). The rod opsin gene was shown to be fully functional as demonstrated by the recovery of the rhodopsin pigment from transiently transfected mammalian COS-1 cells (Oprian et al. 1987). This was the preferred method for preparing rhodopsin mutants when I joined the laboratory in early 1994. African green monkey COS-1 cells were transfected with pMT3-based plasmids by using DEAE-mediated transfection. The synthetic bovine rod opsin gene in this expression plasmid is under control of the SV40 major late promoter. In a standard experiment, COS-1 cells were harvested 60–72 h after transfection by scraping cell monolayers from the surface culture dishes. This step is particularly labour intensive and time consuming and is one of the bottlenecks for generating large amounts of rhodopsin pigment. The harvested cells were treated with 11-*cis* retinal in the dark to form the rhodopsin pigment prior to solubilisation with detergent (Oprian et al. 1987). A single-step small-scale immunoaffinity purification system was developed (Oprian et al. 1987), which was later optimised to enable the separation of misfolded rod opsin populations and correctly folded rhodopsin (Ridge et al. 1995). This transient transfection method of transfection is highly effective for the rapid production of 10–100 ug amounts of purified rhodopsin mutants, and production is scaled up by increasing the number of dishes of cells transfected. This method is ideal and sufficient for preparing samples of rhodopsin mutants for use in UV–vis spectroscopy, signal transduction assays, and fluorescence spectroscopy experiments. However, this transfection method is time consuming, labour intensive, and expression levels were inconsistent. As a consequence expression levels were relatively low and variable, typically ranging from 7 to 15 ug per 15 cm cell culture dish (about 1–3 × 10^7^ COS-1 cells). These low expression levels meant that many powerful but insensitive biophysical methods such as FTIR (1 mg levels) and NMR (3–5 mg levels) were impractical. For this reason, several other expression systems (e.g., *Escherichia coli*, baculovirus infected insect cells, yeast, and cell free systems) remained under investigation in the Khorana laboratory, but progress for most remained limited and much of this work was never published.

**Fig. 1:**
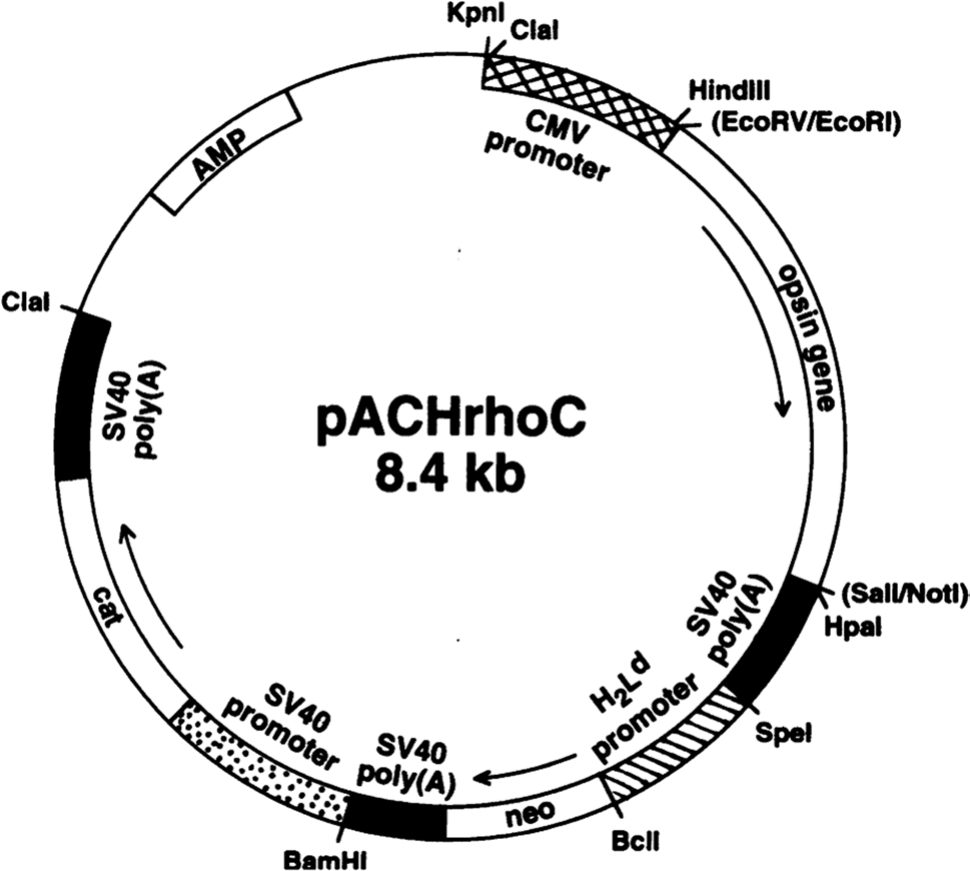
Expression vector pACHrhoC for construction of mammalian stable cell lines. The rhodopsin gene is controlled by the strong constitutively active CMV promoter. This plasmid also contains the gene for G418 resistance which is under control of the weak H_2_L^d^ promoter. Integration of this plasmid into transcriptionally active regions of the chromosome is selected by growth of transfected HEK293S cells in the presence of high concentrations (2–3 mg/ml) of G418. Further details for construction and use of this plasmid are described in the original manuscript (Reeves et al. 1996). Copyright (1996) National Academy of Sciences, USA

**Fig. 2:**
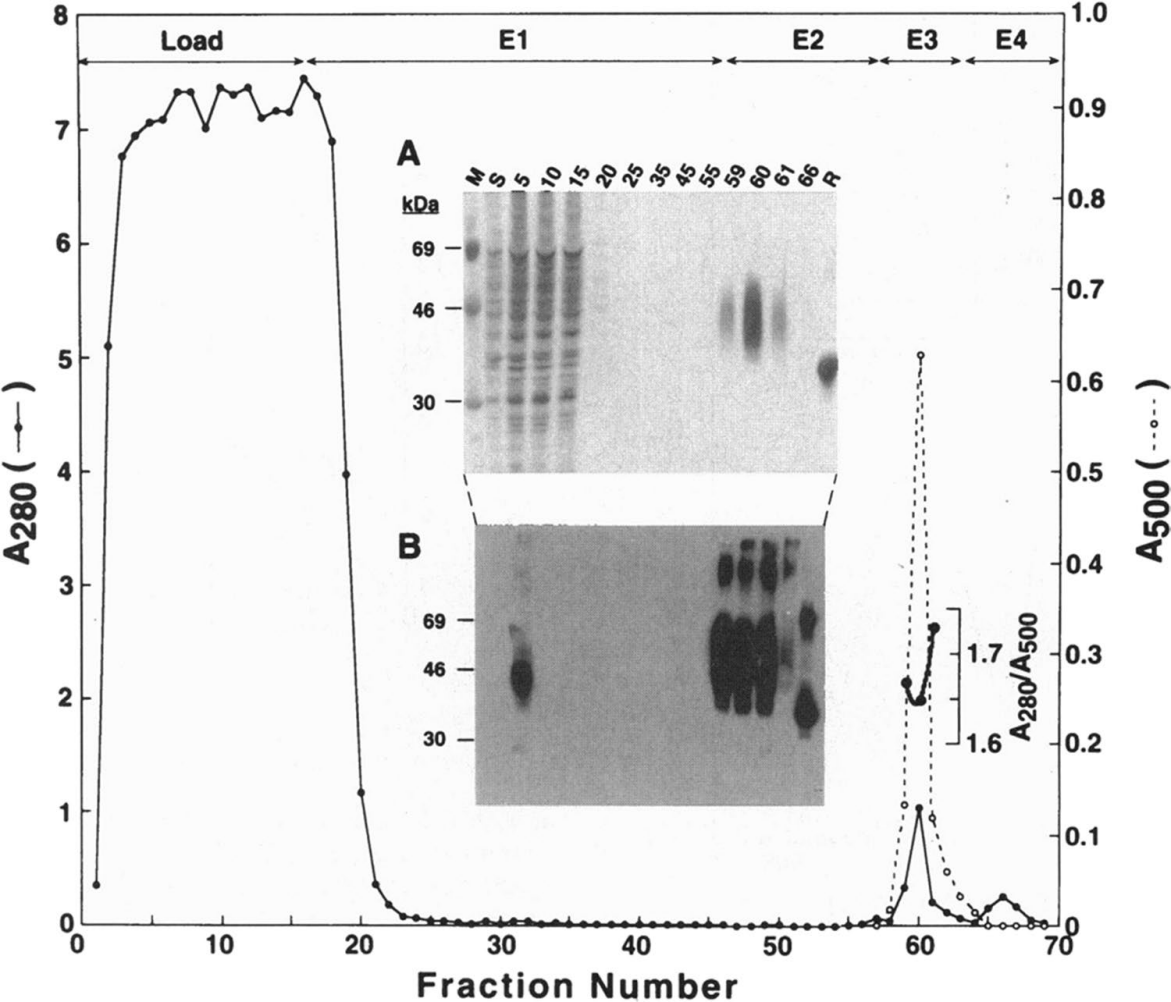
Immunoaffinity purification of a milligram of rhodopsin from a HEK293S stable cell line grown in suspension culture. A cell line stably transfected with pACHRhoC (Fig. 1) was grown in suspension culture (550 ml). Rhodopsin was purified from harvested cells by a single-step immunoaffinity procedure using Rho-1D4 Sepharose. Fractions were collected and analysed during the entire procedure. After loading the column (load) the column was washed with PBS (E1), a low salt buffer (E2), the same low salt buffer containing the 9mer elution peptide (E3) and finally PBS containing the 9mer elution peptide (E4). Correctly folded rhodopsin (*A*_280nm_/*A*_500nm_ ratio of ~ 1.65) elutes sharply from this column from fractions collected using buffer E3. SDS-PAGE analysis followed by silver staining (A) and Western blot (B) is shown as an inset. Full details of this experiment are described in Reeves et al. (1996) from which this figure was reproduced. Copyright (1996) National Academy of Sciences, USA

When I started in the Khorana laboratory—at that time located in the Chemistry building 18 at MIT—I joined a project that was exploring the use of mammalian stable cell lines to express the rhodopsin gene. Gobind was confident for the potential of this approach based on his discussions with Jeremy Nathans who had previously published work describing the construction of suspension-adapted human embryonic kidney (HEK293S) cell lines expressing WT and mutant forms of rhodopsin (Nathans et al. 1989). In early 1995 I contacted Jeremy Nathans to request the HEK293S cell line along with an HEK293S stable cell line expressing rhodopsin. These cell lines were kindly supplied but unfortunate, the expression vector used by Jeremy Nathans for making the stable cell lines was not readily available. I contacted several other research groups in order to test their expression plasmids, often designed for expression of other eukaryotic genes. The plasmid that gave rise to HEK293S stable cell lines expressing the highest level of rhodopsin was pACHEnc which was designed for expression of acetylcholinesterase (Kronman et al. 1992). This vector (Fig. 1) contains the strong CMV promoter for high-level expression of the target gene along with a selectable marker (Neo) driven by a relatively weak promoter (H_2_L^d^). The rationale behind this design is that integration of this plasmid into chromosomal locations favourable for high transcriptional activity is more likely to confer resistance to the selection antibiotic G418, especially when used at high concentrations. In our experiments, stable cell lines were selected using G418 concentrations of up to 2 mg/ml in the growth medium. The expression level of rhodopsin in one of the cell lines obtained was in the range of 50 ug from a confluent 15 cm cell culture dish (Reeves et al. 1996). This value is three to seven times higher than that achieved by transient transfection of COS-1 cells, as described previously. Attention was then directed to the optimisation of growth of cell lines in suspension. We also had to consider the scale-up of immunoaffinity purification using a column format, characterisation of the rhodopsin made by HEK293S, and demonstrating applicability of the system by making and characterising rhodopsin mutants. Gobind offered valuable support and input during the development of these methods as did Tom RajBhandary and members of both research groups. One of our landmark results was the single-step purification of > 1 mg of rhodopsin from a 550-ml suspension culture of an HEK293S cell line containing an integrated rod opsin gene (Fig. 2) (Reeves et al. 1996). A most memorable Friday research group meeting was one with Gobind’s former colleagues, Mike Smith, in attendance. I presented our work on rhodopsin stable cell lines at that meeting and it was clear that Gobind was immensely proud of the progress we were making. This first work on the HEK293S expression system was completed in the following 12 months and published in October 1996 (Reeves et al. 1996). Writing this manuscript with Gobind was an unforgettable and enjoyable experience which often included meetings on Saturday morning. Here I experienced Gobind’s cut and paste editing method for preparing manuscripts. Using scissors Gobind would carefully cut out passages of my typed text and stick them onto A4 paper with Sellotape. Gaps were left for Gobind to insert his hand-written text. Once this new draft was assembled, it was handed over to Gobind’s secretary, Judy Carlin, who would type up the next version. This cut and paste process would be repeated at the next meeting. Figures for manuscripts, for example, Figs. 1 and 2 in this letter, were prepared by Gobind’s daughter, Julia, who is a talented graphic designer. Attention to detail was absolute through the entire manuscript writing process and we would often spend several hours perfecting a single figure. Gobind would identify the best example of a figure from his vast collection of previously published papers which were bound into volumes and stored in his office. His incredible memory was such that he knew exactly which paper, from a collection of over 600, that he needed to identify. It was especially during these times of writing manuscripts that I got to know Gobind well and I learned more about his interests, for example, in coin collecting and in classical music.

The HEK293S stable cell line expression system was first used to investigate rhodopsin containing mutations in the cytoplasmic loops and their interactions with rhodopsin kinase (Thurmond et al. 1997). In 1999 we reported the use of this system to incorporate stable isotope labelled amino acids into rhodopsin for magic angle spinning NMR studies (see next section). These experiments and others were often performed using rhodopsin prepared from the HEK293S cell lines grown to saturation in 500 ml culture volumes. These suspension cultures were grown in multiple slowly stirred 2-l spinner flasks. While generally successful, the growth conditions used were sometimes unreliable and at times cultures failed to grow. For certain rhodopsin mutants, we were unable to obtain stable cell lines, and this was also a problem in our attempts to make stable cell lines expressing rhodopsin kinase. Discussions with Gobind, especially during group meetings, inspired the development of procedures to tackle these new challenges. Gobind offered the academic freedom and advice necessary to overcome these and many other challenges.

## Comparison of the expression systems for rhodopsin examined in the Khorana laboratory

In order to identify the most suitable expression host for a particular gene, it would be useful to compare experimental outcomes side by side. This is often not possible because of the very different expertise required to handle the various expression hosts. The expression of the synthetic rod opsin gene in the Khorana laboratory made such comparisons possible. Indeed, we have a direct comparison with a yeast *Saccharomyces cerevisiae* expression system which was under investigation at the same time by a PhD student in the lab, Ramin Mollaaghababa. Ramin and I performed many of the experiments on rhodopsin made in yeast and by HEK293S stable cell lines in parallel. Milligram amounts (2.0 mg/3.7 l culture) of rhodopsin were made by yeast under the conditions reported; however, only a small fraction (3–4%) could form rhodopsin upon treatment with 11-*cis* retinal (Mollaaghababa et al. 1996). Despite this limitation it was still possible to purify 134 µg of pure and functional rhodopsin using the separation method previously optimised by Kevin Ridge (Ridge et al. 1995). Results similar to those in the Khorana laboratory were obtained by Kevin Ridge’s group using the methylotrophic yeast *Pichia pastoris* (Abdulaev et al. 1997). Expression and purification of rhodopsin using Xenopus oocytes was also attempted with purification yields in the 10 µg range (Khorana et al. 1988). Attempts were made to express rhodopsin in baculovirus-infected Sf9 insect cells (S. Kaushal PhD, thesis). This work was done in the early 1990s where this expression system was less well developed and more difficult to work with. While pure functional rhodopsin was obtained, much of the opsin produced did not form pigment when treated with 11-*cis* retinal. The problems associated with rhodopsin misfolding in these different expression systems might have been surmounted if rhodopsin pigment could have been generated from the misfolded forms, in a manner similar to bR, which could be refolded from the denatured state. However, despite intense effort by A. Kronis and Khorana, purified bovine rhodopsin once denatured could not be re-folded (described in Sakamoto and Khorana (1995)). Indeed, rod opsin derived from light-activated rhodopsin had a time–restricted capacity to reform pigment in the dodecyl maltoside detergent environment (Sakamoto and Khorana 1995).

A summary of the challenges encountered in the attempts to produce large quantities of folded and functional rhodopsin as well as the limitations of alternative systems was outlined in our manuscript published in 1986 (Reeves et al. 1996); the following passage was written:

“Considerable effort has indeed been expended in a number of laboratories on investigating different expression systems for the preparation of opsin and its mutants. Expression in *E. coli* has not been encouraging (S. Karnik and P. Loewen, unpublished work in this laboratory). The yeast system, *S. cerevisiae*, as well as the methylotrophic* Pichia pastoris*, have been investigated recently (Mollaaghababa et al. 1996), but have not resulted in any significant advance for larger scale preparation of fully functional protein. The frequently used baculovirus-based expression system has been investigated by DeGrip and colleagues (Decaluwe et al. 1993, Janssen et al. 1995, 1990) and in this laboratory (S. Kaushal, unpublished work). In our view, this system has not resulted in any improvement in the availability of fully functional post-translationally processed protein that can be reliably used in signal transduction studies.”

## Application of the HEK293S expression system for NMR studies of rhodopsin

In 1998 a collaboration with the research group led by Steve Smith (then at Yale) was initiated in an attempt to prepare samples for Solid State MAS NMR spectroscopy experiments. Markus Eilers from Steve Smith’s lab visited MIT during the summer of 2008 to do this work. The challenge was to purify milligram amounts of rhodopsin incorporating stable isotope-labelled amino acids. To do this we first had to formulate calcium-free DMEM growth medium so that specific stable isotope labelled amino acids could be added. At this point in time, we were using the HEK293S stable cell line constitutively expressing WT rhodopsin (Reeves et al. 1996) and we grew multiple 500-ml suspension cultures in 2-l volume spinner flasks. Milligram amounts of 6-^15^N-lysine and 2-^13^C-glycine were thus prepared and used in solid state MAS NMR experiments. These experiments were successful, and we were able to observe the Schiff-base lysine resonance (Eilers et al. 1999) and determine the distance between the retinal K296 protonated Schiff base and the E113 Schiff base counterion. This result, before publication of the rhodopsin crystal structure, demonstrated the feasibility of this approach for probing structure and activation of rhodopsin. However, Gobind was very aware of the limitations of this expression system, and this illuminated the path we needed to take to further refine this system. This is evident in the discussion section of this paper which contains the following passage:

“This system has provided the amounts of the receptor that are required for biophysical studies, such as those involving MAS NMR. Although the amounts obtained in this study are adequate, clearly more effort is possible in scaling up rhodopsin production by further optimizing cell culture conditions, receptor purification, and incorporation into membranes.”

This passage clearly demonstrates Gobind’s scientific thinking and vision. While the expression system we had developed was satisfactory and was also deployed successfully for ^19^F solution NMR studies of rhodopsin (Klein-Seetharaman et al. 1999), there remained much scope for improvement. Some of the further developments that aimed to address these limitations will be described below.

## Growth of HEK293S cell lines in suspension culture using a bioreactor

Much of the research on bR conducted in the Khorana was performed using bR mutants expressed in *E. coli* grown in a bacterial fermenter. It was Gobind’s idea to explore the equivalent format for growing large amounts of mammalian cell lines in suspension culture. By this time, we had gained adequate expertise for the routine growth of HEK293S cell lines using spinner flasks to make rhodopsin mutants. Exploration of growth of these cell lines in a bioreactor was the next reasonable step. Unfortunately, information regarding the culture of HEK293S cells in such a format was not available. For this reason, Gobind encouraged me to visit other laboratories to test the suitability of their facilities for growth of our HEK293S cell lines. My first visit was to Dan Oprian’s laboratory nearby in Brandeis University. Dan Oprian’s group used an Applikon bioreactor mainly for growth of hybridomas, but unfortunately this format was not optimal for HEK293S cells. I next visited the research centre of New Brunswick Scientific in Edison (NJ) to perform a growth experiment. Preliminary results were favourable, so we invested in the Celligen Plus bioreactor equipped with a pitch blade impeller. The optimised growth of HEK293S cell lines in this system coupled with the tetracycline inducible technology described below made it possible to prepare rhodopsin mutants in 10 mg amounts from culture volumes of about 1 l (Reeves et al. 2002b). This level of expression was sufficient for the preparation of samples for several subsequent NMR studies.

## Further improvements to the HEK293S stable cell line expression system in the Khorana laboratory

**Fig. 3:**
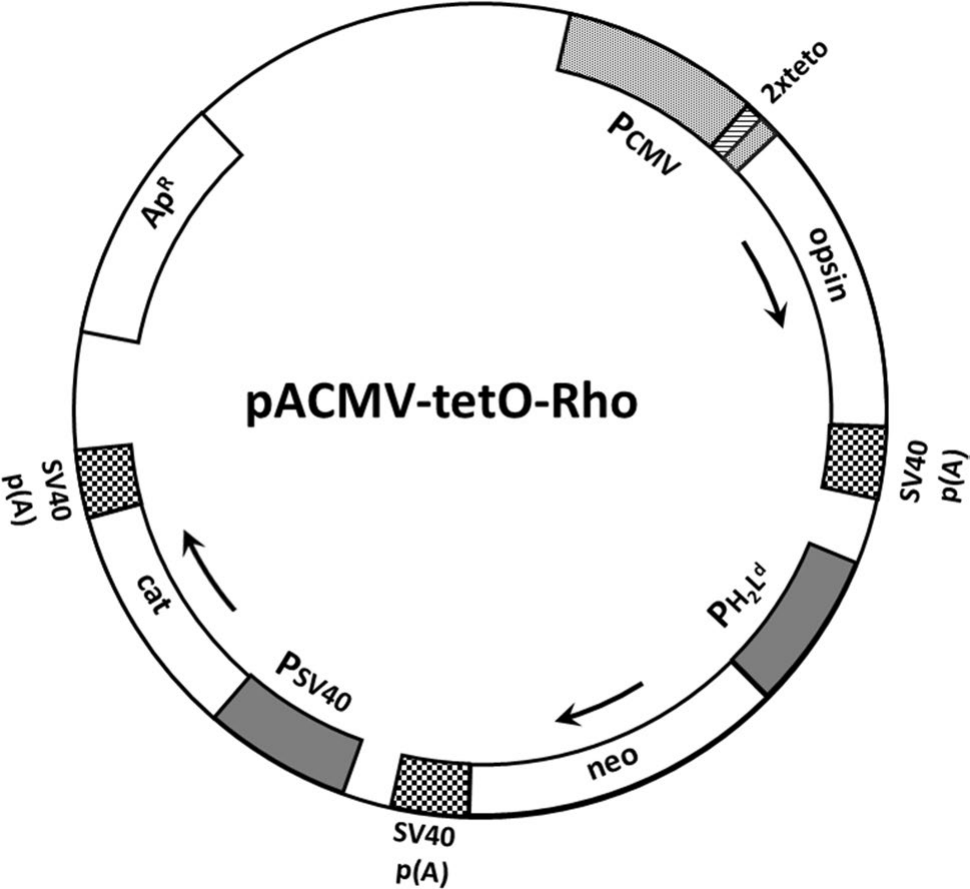
Expression vector pACMV-tetO-Rho for construction of inducible mammalian stable cell lines. This expression plasmid carries the bovine rhodopsin gene under the control of a human CMV promoter containing two tandem tetO DNA sequences. When this plasmid is integrated into the genome of HEK293S cells expressing the tetracycline repressor protein (TetR), expression from this hybrid CMV promoter is blocked. Stable cell lines transfected with this plasmid are obtained by using G418 selection. Optimal inducible expression from the CMV-tetO promoter is brought about by addition of tetracycline and sodium butyrate to the growth medium followed by further incubation for 60–72 h. Details for construction of this plasmid have been described previously (Reeves et al. 2002b) and this figure is reproduced from Fig. 1 in that publication. Copyright (2002) National Academy of Sciences, USA

The stable expression system we favoured thus far used the strong constitutive human cytomegalovirus promoter for expression of the rhodopsin gene. However, our attempts to generate stable cell lines expressing certain rhodopsin mutants such as the constitutively active triple mutant (E113Q/E134Q/M257Y) as well as rhodopsin kinase both failed. Both of these proteins could be prepared in transient systems which led us to believe that there could be problems associated with cytotoxicity of these gene products when expressed constitutively. At about this time a research paper was published describing the development of a tetracycline inducible expression system (Yao et al. 1998). This system utilised a full length human CMV promoter modified to contain two tandem transposon tn10 operator sequences towards the 3′ end. In this tetracycline-regulated expression system, the CMV promoter is controlled in a manner reminiscent of the classical bacterial operon. The key features of this plasmid are described in Fig. 3. This is different in design to the more popular tetracycline inducible control system developed by Bujard’s group in which a split transactivator is used to control gene expression (Gossen and Bujard 1992). We used the system designed by Yao et al. (1998) and combined it with elements from pACHEnc vector as described by us previously (Reeves et al. 1996). A plasmid carrying the gene encoding the tetracycline repressor protein (TetR) was first used to make the cell line HEK293S-TR which makes constitutively the TetR repressor protein. The construction of stable cell lines using this tetracycline inducible system led to a 2- to tenfold increase in rhodopsin expressed upon induction compared to our constitutive stable cell lines (Reeves et al. 2002b). These results would reasonably suggest that our original constitutive expression system had an upper limit for expression of receptors. The tightly controlled regulation afforded by the tetracycline inducible system overcame this limitation and enabled the construction of cell lines encoding genes that were cytotoxic and the potential for much higher levels of expression. This tetracycline inducible expression system has been used by us since then for the production of stable isotope-labelled rhodopsin for use in MAS NMR studies to probe light activation (Patel et al. 2005). We are still using this system developed in the Khorana laboratory for our research but we are mindful of a major limitation—the long time needed for generating stable cell lines (about 8–10 weeks).

## Complex N-glycosylation of rhodopsin produced in HEK293S cell lines and isolation of a variant for production of glycoproteins containing simple N-glycans

**Fig. 4:**
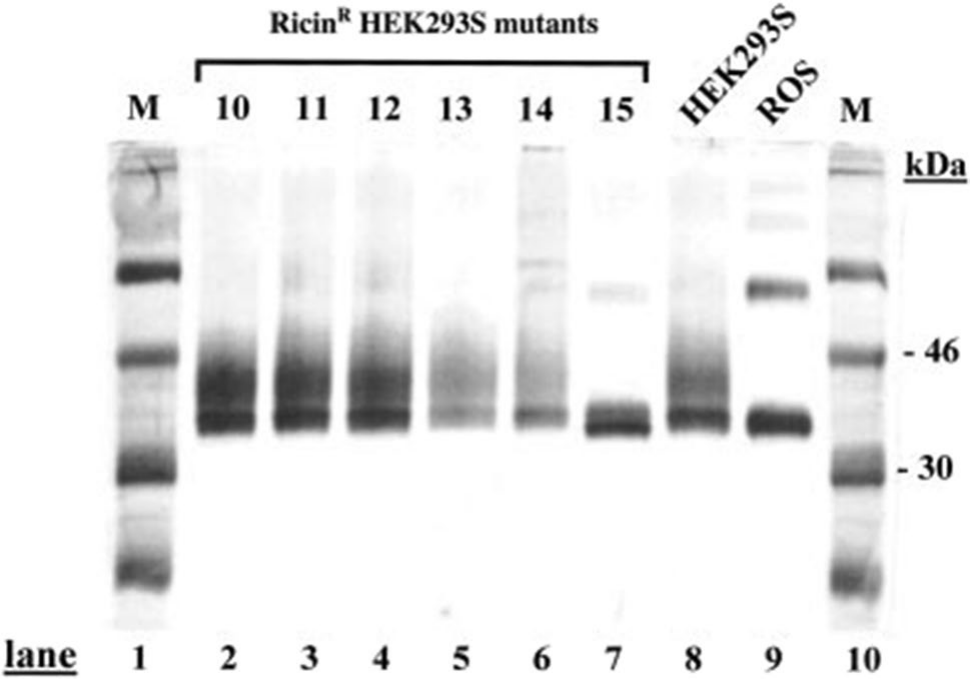
Isolation of a HEK293S cell line for production of glycoproteins containing short homogeneous N-glycans. HEK293S cells were subjected to chemical mutagenesis followed by growth in the presence of ricin to select for cell lines defective for complex N-glycan synthesis. Surviving HEK293S cell lines resistant to ricin were expanded and transfected with pMT4-Rho. Rhodopsin produced was purified and examined by SDS-PAGE followed by silver staining. Lanes 8 and 9 contain the control HEK293S and bovine retina rhodopsin, respectively. Rhodopsin purified from ricin-resistant HEK293S cell line candidate 15 (lane 7) no longer has a high molecular weight trailing smear but instead migrates like rhodopsin purified from bovine retinas. This image is from Reeves et al. (2002a). Copyright (2002) National Academy of Sciences, USA

We were interested in producing rhodopsin samples for use in crystallisation trials, especially using a photobleaching resistant mutant (Ridge et al. 1992). However, crystallographers were concerned about the microheterogeneity of the purified rhodopsin as observed on SDS-PAGE (Reeves et al. 1996). We were successful in attempts to deglycosylate rhodopsin by growth of cell lines in tunicamycin but results were inconsistent (Reeves et al. 2002b). I was fortunate to meet Roland Contreras, a former colleague of Gobind, at the Khorana symposium held in Brandeis during the summer of 2000. Roland had spent much of his career working on glycosylation and it was he who suggested we should try to isolate complex glycosylation defective mutants of HEK293S cells using growth in the presence of lectins as selection. A similar strategy had been used previously to make mutants of CHO cells defective in various glycosylation modifications (Stanley and Chaney 1985; Stanley et al. 1990). HEK293S cells were mutagenised using EMS and then grown under selection with various lectins. After 2–3 weeks a few colonies of HEK293S cells resistant to RCAII (ricin) were isolated and expanded. Further experiments in collaboration with Nico Callewaert and Roland Contreras demonstrated that opsin transiently expressed in one of these cell lines had simple N-glycosylation profile (GlcNAc_2_Man_5_) compared to the parental line (Reeves et al. 2002a). In Fig. 4 the SDS-PAGE profile of HEK293S mutant candidates transfected with a plasmid containing the opsin gene (Oprian et al. 1987) is shown. Lane 7 in this figure shows that rhodopsin produced by this mutant cell line migrates similarly to that of rhodopsin purified from bovine retinas (lane 9) and no longer has a trailing smear observed in rhodopsin produced by parental HEK293S cells (lane 8). N-glycan profiling of these rhodopsin samples corroborated these findings (Reeves et al. 2002a). We next demonstrated that this line was defective for N-acetylglosamine transferase I activity, a result that explained the ricin-resistant phenotype and the N-glycan profile. In combination with a thermally stabilised mutant of rhodopsin (Xie et al. 2003), this HEK293S GnTI^−^ cell line was used for rhodopsin crystallisation (Standfuss et al. 2007) and it is now one of the most popular cell lines used to make proteins for use in structural biology (Bussow 2015). After I presented this work at a Monday seminar, Tom RajBhandary suggested we should make an application to patent this cell line. Gobind agreed but he was far more interested in publishing this work and making the components accessible to the scientific community. The final part of this research project was to assemble the tetracycline inducible system into this HEK293S GnTI^-^ cell line as described above. This cell line grew well in bioreactor suspension culture and upon induction made rod opsin in the 5–10 mg/l range (Reeves et al. 2002a). Several years later this HEK293S GnTI^-^cell line was subjected to whole genome sequencing. The expectation was to find point mutations, but instead whole deletions of chromosome locations of the GnTI loci were found (Lin et al. 2014).

## Future technical developments for the high-level expression of rhodopsin and other GPCRs in mammalian systems

While the baculovirus/Sf9 system has proven to be the most effective system for producing GPCRS in large amounts and of sufficiently high quality for the majority of structural investigations (Lv et al. 2016), there remains the likelihood that certain GPCRS will not express well or will fold incorrectly in insect cells. Rhodopsin is a prime example of a receptor that folds poorly or is modified incompletely in this insect cell system. It is therefore prudent to continue to develop and improve alternative expression systems such as those based on the human HEK293S cell line. The main limitation of the current HEK293S stable line system is the length of time and intensity of labour required to isolate high-level expressing cell lines. The Flp-in system is one such development which allows targeted integration of target genes a single site, often referred to as a landing site, of the genome that is highly transcriptionally active (Ward et al. 2011). Presumably this removes or reduces the need for screening numerous colonies in order to identify the best cell lines. Large-scale transfection using plasmid-PEI complexes (Cervera and Kamen 2018) or infection with virus vectors (Elegheert et al. 2018; Goehring et al. 2014) of HEK293S cells growing in suspension culture has also been developed to bring the HEK293S system closer to the baculovirus/sf9 system in terms of speed and simplicity. Another promising approach is the development of transposon-based integration of genes into the chromosome. This procedure has the potential for efficient integration of target genes into transcriptionally active loci and in multi copy, thus increasing gene dosage. There is therefore the potential for rapid construction of regulated gene expression constructs using pools of cell lines (Caro et al. 2015). It is this approach that we are now exploring in order to overcome this important remaining bottleneck.

## Data Availability

Not applicable to this article as no datasets were generated or analysed during the current study.
